# Subclinical tuberculosis linkage to care and completion of treatment following community-based screening in rural South Africa

**DOI:** 10.1186/s44263-024-00059-0

**Published:** 2024-06-02

**Authors:** Zolelwa Sifumba, Helgard Claassen, Stephen Olivier, Palwasha Khan, Hloniphile Ngubane, Thokozani Bhengu, Thando Zulu, Mareca Sithole, Dickman Gareta, Vukuzazi Team, Mahomed-Yunus S. Moosa, Willem A. Hanekom, Ingrid V. Bassett, Emily B. Wong

**Affiliations:** 1 Africa Health Research Institute, Durban & Somkhele, KwaZulu-Natal South Africa; 2 Department of Infectious Disease, Nelson R Mandela School of Medicine, University of KwaZulu-Natal, Durban, South Africa; 3 London School of Hygiene & Tropical Medicine, Department of Clinical Research, Faculty of Infectious and Tropical Diseases, London, Great Britain and Northern Ireland UK; 4 King Edward VIII Hospital, Durban, South Africa; 5 Division of Infection and Immunity, University College London, London, Great Britain and Northern Ireland UK; 6 Division of Infectious Diseases, Massachusetts General Hospital, Boston, MA USA; 7 Division of Infectious Diseases, University of Alabama Birmingham, Birmingham, AL USA

**Keywords:** Tuberculosis, Asymptomatic, Subclinical TB, Linkage, Cascade of care

## Abstract

**Background:**

Tuberculosis (TB), a leading cause of infectious death, is curable when patients complete a course of multi-drug treatment. Because entry into the TB treatment cascade usually relies on symptomatic individuals seeking care, little is known about linkage to care and completion of treatment in people with subclinical TB identified through community-based screening.

**Methods:**

Participants of the Vukuzazi study, a community-based survey that provided TB screening in the rural uMkhanyakude district of KwaZulu-Natal from May 2018 – March 2020, who had a positive sputum (GeneXpert or Mtb culture, microbiologically-confirmed TB) or a chest x-ray consistent with active TB (radiologically-suggested TB) were referred to the public health system. Telephonic follow-up surveys were conducted from May 2021 – January 2023 to assess linkage to care and treatment status. Linked electronic TB register data was accessed. We analyzed the effect of baseline HIV and symptom status (by WHO 4-symptom screen) on the TB treatment cascade.

**Results:**

Seventy percent (122/174) of people with microbiologically-confirmed TB completed the telephonic survey. In this group, 84% (103/122) were asymptomatic and 46% (56/122) were people living with HIV (PLWH). By self-report, 98% (119/122) attended a healthcare facility after screening, 94% (115/122) started TB treatment and 93% (113/122) completed treatment. Analysis of electronic TB register data confirmed that 67% (116/174) of eligible individuals started TB treatment. Neither symptom status nor HIV status affected linkage to care. Among people with radiologically-suggested TB, 48% (153/318) completed the telephonic survey, of which 80% (122/153) were asymptomatic and 52% (79/153) were PLWH. By self-report, 75% (114/153) attended a healthcare facility after screening, 16% (24/153) started TB treatment and 14% (22/153) completed treatment. Nine percent (28/318) of eligible individuals had TB register data confirming that they started treatment.

**Conclusions:**

Despite high rates of subclinical TB, most people diagnosed with microbiologically-confirmed TB after community-based screening were willing to link to care and complete TB treatment. Lower rates of linkage to care in people with radiologically-suggested TB highlight the importance of streamlined care pathways for this group. Clearer guidelines for the management of people who screen positive during community-based TB screening are needed.

**Supplementary Information:**

The online version contains supplementary material available at 10.1186/s44263-024-00059-0.

## Background

Tuberculosis (TB) is a leading infectious disease cause of death globally [1] and the leading cause of death for people living with HIV (PLWH) in South Africa [2]. TB is eminently curable but successful management requires engagement in an entire care cascade from diagnosis to completion of treatment [3]. In many countries, including South Africa, many patients fall out during this cascade, resulting in suboptimal treatment, increased morbidity, mortality, drug-resistance and onward transmission.

Diagnosis, the beginning of the care cascade, has traditionally relied on symptom-based screening [4]. However, there is increasing recognition that subclinical disease, in which a patient has radiological or microbiological evidence of TB with minimal or no symptoms, could be a contributing factor to the global burden of disease [5]. It is estimated that approximately half of prevalent TB is asymptomatic [5], which fails to trigger symptom-driven diagnostic testing and may serve as a reservoir for ongoing transmission in the community [6].

Little is known about the experiences of people diagnosed with subclinical, or asymptomatic TB, during community-based screening: their likelihood of linking to care, rates of treatment commencement and completion, and health outcomes. There are currently no guidelines to assist healthcare providers in decisions about treatment of these patients [7]. Care pathways for patients whose screening indicates radiological signs of active TB without microbiological confirmation are especially complex, since it requires additional diagnostic and clinical evaluations before a decision can be made whether or not to start TB treatment [8, 9].

Our objective was to understand linkage to TB care, including starting and completing treatment, among patients diagnosed with microbiologically-confirmed or radiologically-suggested TB during community-based screening in a rural South African setting, and to determine if baseline symptom status and HIV status played a significant role in the cascade of care.

## Methods

### Study setting

**Fig. 1:**
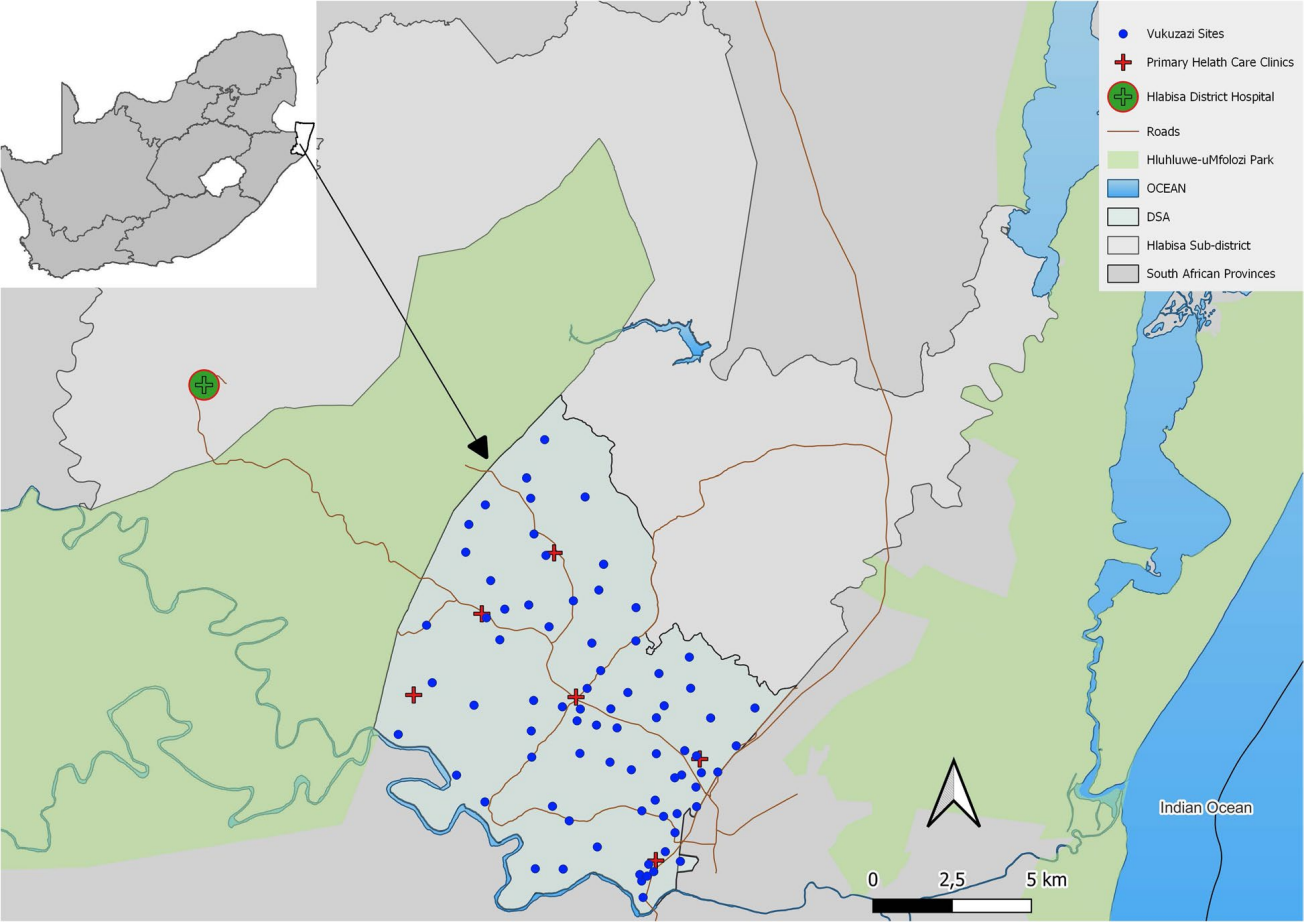
AHRI demographic surveillance area with sites of Vukuzazi community-based screening in relation to Department of Health facilities. Blue dots = health screening camps, Red crosses = primary health care clinics, Green circle = district hospital

The study took place at the Africa Health Research Institute (AHRI) demographic surveillance area (DSA), which is located in the rural uMkhanyakude district in KwaZulu-Natal, South Africa (light blue in Fig. 1). The southern section of the AHRI DSA was targeted, an area of 438km^2^ with a population of approximately 65,000 [10]. This community has one of the highest HIV prevalence rates in the world: 34% in the general population and 62% among women aged 25–44 years [11]. Six primary health care clinics are located in the southern AHRI DSA. The closest hospital is Hlabisa District Hospital, which is 23.5km away by road from the western edge of the area. Demographic and health surveillance, including record of deaths, is conducted yearly in this area.

### Community-based screening: study procedures, definitions, referral for care

#### Study procedures

Eligible adolescent and adult residents aged 15 years and older from the southern DSA were invited to participate in a multi-disease community-based screening program called ‘Vukuzazi’ (which means “wake up and know yourself” in isiZulu) from May 2018 to March 2020. Individuals were visited at their homes and invited to attend a mobile health camp that moved through the study area during the study period (dark blue dots in Fig. 1). Screening activities included tests for TB, HIV, elevated blood pressure and elevated blood glucose. The detailed methods of the survey have been described by Gunda et al. [10]; here, HIV and TB screening procedures are summarized. Research nurses conducted standardized interviews including questions about HIV history and treatment and current and previous TB diagnoses and treatment. To assess TB symptoms, participants were asked whether they currently had any of World Health Organization’s TB 4-symptom screening (WHO-4SS) questions: cough (of any duration), fever, night sweats or unintentional weight loss (over the last 6 months) [4]. All non-pregnant participants underwent posterior–anterior digital mobile chest X-ray, with automated assessment by computer-aided detection for TB (CAD4TB, Delft Imaging, Hertogenbosch, The Netherlands) software and off-site interpretation by a specialist radiologist with more than 35 years of experience. The radiologist, who was blinded to all clinical information and CAD4TB version 5 (CAD4TBv5) score, designated each image as having either normal or abnormal lung fields and classified abnormalities according to the WHO’s Tuberculosis Prevalence Surveys handbook [12] which included a category “ADS-TB: Abnormality detected, significant – tuberculosis” (details in Additional file 1: Supplementary Methods). Any participant who answered yes to any of the WHO-4SS questions, who had a chest x-ray score ≥ 25 (CAD4TBv5), who had abnormal lung fields (as determined by the radiologist), or who was unable to undergo chest x-ray was offered sputum collection. A CAD4TB score reflects the degree of abnormality and likelihood of TB, but there is no universally accepted CAD4TBv5 score to use in a TB screening setting [13]. In a process previously detailed by Fehr et al. [14], a CAD4TBv5 score of 25 threshold was selected to maximize TB case-finding in this study. Sputum was tested for *Mycobacterium tuberculosis* (Mtb) with an Xpert MTB/RIF Ultra test (Cepheid, Sunnyvale, CA, USA) and liquid mycobacterial culture (BACTEC MGIT 960 System (Becton Dickinson, Berkshire, UK)) [11]. Blood was obtained to assess HIV status (Genscreen Ultra HIV Ag-Ab enzyme immunoassay (Bio-Rad)); participants with a positive HIV immunoassay always had a reflex HIV-1 RNA viral load performed (Abbott RealTime HIV-1 Viral Load (Abbott, IL, USA)).

#### Definitions

According to pre-specified definition, individuals who were not receiving TB treatment at the time of the survey and whose sputum was positive for Mtb by either Xpert Ultra (including trace) and/or Mtb culture, were considered to have newly diagnosed microbiologically-confirmed TB. Individuals who were not receiving TB treatment at the time of the survey and whose sputum was negative for Mtb but whose chest x-ray was interpreted by the radiologist to be consistent with active TB (categorized as ADS-TB: abnormality detected, significant – tuberculosis), were considered to have radiologically-suggested TB. Participants with TB were classified as symptomatic if they had any of the WHO-4SS symptoms and asymptomatic if they had none of the WHO-4SS symptoms. Participants who had microbiologically-confirmed TB and were asymptomatic at baseline met the study definition for subclinical TB. HIV status was defined as positive or negative based on the HIV immunoassay result.

#### Referral to care

Individuals with newly diagnosed microbiologically-confirmed or radiologically-suggested TB were informed of their results by a research nurse during a home visit, provided documentation of their results and a printed copy of their chest x-ray and referred for further care to the nearest primary health care clinic or hospital. Further management was conducted by the local department of health (DoH) personnel according to standard protocols. Exceptions to standard protocols in management were documented in a memorandum of understanding (MOU) signed by AHRI and the local DoH prior to starting the study. This document stated that positive Xpert Ultra results would be considered as a microbiological diagnosis of TB disease even if subsequent sputum tests collected in DoH clinics and tested by the National Health Laboratory Service (NHLS) were negative. The MOU also stated that patients with microbiologically-confirmed TB diagnosed by AHRI would be referred to the nearest DoH nurse-led primary health care clinic within 72 h and that the DoH would be provided the names and addresses of individuals from this category who failed to link to care within 72 h. Because the evaluation of radiologically-suggested TB required interpretation of the chest x-ray and clinical assessment, these patients were referred directly to the district hospital for physician evaluation.

### Assessment of Linkage to Care: study procedures and definitions

#### Telephonic survey

Vukuzazi participants who met the criteria for either microbiologically-confirmed or radiologically-suggested TB in the baseline survey were reapproached and offered participation in a follow-up telephonic survey between May 2021 – January 2023. Those whose death was confirmed by demographic surveillance data (accessed October 2022), who were unable to be contacted on the telephone after 3 attempts, or who were contacted but were unable or unwilling to consent to spend 15–20 min on the phone to complete the survey were excluded from this study component. Using a structured questionnaire, participants were asked whether they linked to a clinic or hospital for care for TB after Vukuzazi screening and whether they started and completed TB treatment. Based on answers to the telephonic survey, participants were determined to meet the following primary outcomes: “Linked to care”, defined as a self-reported visit to the clinic/hospital as a result of the Vukuzazi screening test within 90 days; “Started TB treatment”, defined as self-reported starting of TB treatment within 90 days of the Vukuzazi screening date; “Completed TB treatment”, defined as self-reported completion of 6 months of treatment for drug susceptible-TB within 1 year of screening visit. Time to start and completion of TB treatment were calculated from the date of screening visit.

#### Health data linkage

To mitigate the survivor bias inherent in the data obtained by the telephonic survey, linkage to health system data was attempted for all participants who met the criteria for either microbiologically-confirmed or radiologically-suggested TB in the baseline survey, regardless of vital status. The national DoH Tier.net database (electronic TB register) was queried from the date of Vukuzazi screening to March 2020 for individuals starting treatment using determinative and probabilistic matching based on first name, surname, date of birth, sex and national identification (ID) number. These data resulted in an alternate definition of “Started TB treatment”: starting TB treatment within 90 days of the Vukuzazi screening date based on linkage data obtained from Tier.net. Data after March 2020 were unavailable due to changes in DoH policy in accordance with the national Protection of Personal Information Act (POPIA).

### Data analysis

The sex, age group, residency location, and socioeconomic status of those eligible for each study group and those who completed the telephonic survey were compared. Deaths among eligible individuals were categorized as occurring within the first 90 days after screening and analyzed by baseline symptom status. The percentage of individuals in the microbiologically-confirmed TB and radiologically-suggested TB groups who met the definition of each link in the treatment cascade according to self-report in the telephonic survey was calculated. Data on TB treatment obtained from health system linkage (Tier.net) was assessed for individuals who participated in the telephonic survey and for eligible members of each group. Care cascade and Tier.net data were stratified by baseline symptom and HIV statuses to define the care cascade for people who met the study definition of subclinical TB (asymptomatic microbiologically-confirmed TB) and to assess whether these exposures altered the treatment cascade within each group. Statistical tests used for comparisons between groups included Pearson’s Chi-squared test or Fisher’s exact test for binary data and Wilcoxon rank sum test for ordinal data (specific statistical test used for each comparison indicated in the footnotes of each table). Analyses were done in R statistical software version 4.3.2.

## Results

### Participation in telephonic survey and baseline characteristics

**Fig. 2:**
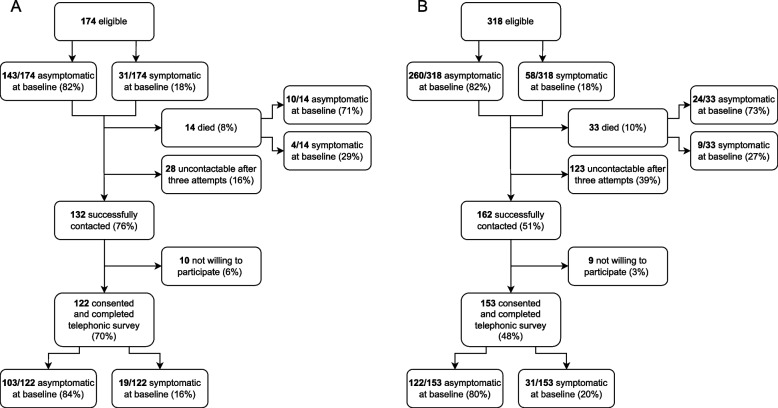
Study inclusion and exclusion pathway in (**A**) the microbiologically-confirmed TB group and (**B**) the radiologically-suggested TB group

174 individuals with microbiologically-confirmed TB and 318 with radiologically-suggested TB were identified, based on baseline screening test results (Fig. 2). Among those with microbiologically-confirmed TB, 14 (8%) had recorded deaths between baseline and follow-up (of which 21% (3/14) occurred within 90 days of baseline screening), 28 (16%) were uncontactable after 3 attempts and 10 (6%) were contacted but unwilling to participate, resulting in 122 (70%) completing the telephonic survey (Fig. 2a). Among those with radiologically-suggested TB, 33 (10%) had recorded deaths between baseline and follow-up (of which 12% (4/33) occurred within 90 days of baseline screening), 123 (39%) were uncontactable after 3 attempts and 9 (3%) were contacted but unwilling to participate, resulting in 153 (48%) completing the survey (Fig. 2b).

Among the 174 individuals eligible for the microbiologically-confirmed TB group, only 31 (18%) were symptomatic at baseline while 143 (82%) were asymptomatic, meeting the study definition for subclinical TB. Similarly, among the 318 individuals eligible for the radiologically-suggested TB group, only 58 (18%) were symptomatic at baseline while 260 (82%) were asymptomatic. Among eligible participants in the microbiologically-confirmed group who died before being able to take part in the survey, 10/14 (71%) were asymptomatic at baseline and 4/14 (29%) were symptomatic. Mortality rates in this group were similar between the symptomatic individuals (4/31 (13%)) and asymptomatic individuals (10/143 (7%), p = 0.3). In the radiologically-suggested group, 24/33 (73%) of those who died were asymptomatic at baseline and 9/33 (27%) were symptomatic. Mortality rates in this group were also similar between symptomatic individuals (9/58 (16%)) and asymptomatic individuals (24/260 (9%), p = 0.2). Among the 122 individuals with microbiologically-confirmed TB who completed the telephonic survey, 103 (84%) were asymptomatic at baseline, meeting the study definition for subclinical TB.

For both microbiologically-confirmed and radiologically-suggested TB, the baseline characteristics – age, sex, TB symptom status, HIV status, residency location, sociodemographic status and CAD4TBv5 score – of people who completed the survey were similar to those of the entire group eligible (Table 1, Part I), with three exceptions. In the microbiologically-confirmed TB group, there were lower rates of participation in the telephonic survey among people of higher socioeconomic status. In the radiologically-suggested group, the 45–64 year old age group was overrepresented among telephonic survey participants, compared with the eligible population and telephonic survey participants had slightly higher CAD4TBv5 scores compared to the eligible population. For clarity, results subsequently presented refer to participants who completed the telephonic survey rather than the larger eligible group, unless otherwise stated. Although the median ages were similar between the microbiologically-confirmed TB patients – 48.5 years (IQR 33–63) – and the radiologically-suggested TB patients – 52 years (IQR 45–63) – the microbiologically-confirmed group was younger with relatively more people aged < 25 years (11% vs. 5%), and fewer aged 45–64 years (32% vs. 56%). Approximately half of participants in both groups were PLWH (46% of those with microbiologically-confirmed TB and 52% of those with radiologically-suggested TB).

**Table 1:** Baseline characteristics, cascade of care and time to TB treatment commencement and completion following community-based screening

	**Microbiologically-confirmed TB**			**Radiologically-suggested TB**			**Comparison between participants from both groups who completed the survey**
	**Eligible**, *N* = 174^a^	**Consented and completed telephonic survey**, *N* = 122^a^	***P*-value**	**Eligible**, *N* = 318^a^	**Consented and completed telephonic survey**, *N* = 153^a^	***P*-value**	***P*-value**
**I. Baseline Characteristics**							
Age group			0.13^2^			0.032^2^	< 0.001^2^
< 25	24 (14%)	14 (11%)		14 (4%)	8 (5%)		
25–44	57 (33%)	42 (34%)		69 (22%)	27 (18%)		
45–64	49 (28%)	39 (32%)		154 (48%)	86 (56%)		
> 65	44 (25%)	27 (22%)		81 (25%)	32 (21%)		
Sex			> 0.9^2^			> 0.9^2^	0.2^2^
Male	80 (46%)	56 (46%)		173 (54%)	83 (54%)		
Female	94 (54%)	66 (54%)		145 (46%)	70 (46%)		
TB symptom status			0.2^2^			0.4^2^	0.3^2^
Symptomatic (WHO-4SS)	31 (18%)	19 (16%)		58 (18%)	31 (20%)		
Asymptomatic (WHO-4SS)	143 (82%)	103 (84%)		260 (82%)	122 (80%)		
HIV ELISA			0.4^2^			0.7^2^	0.3^2^
Negative	98 (56%)	66 (54%)		157 (49%)	74 (48%)		
Positive	76 (44%)	56 (46%)		161 (51%)	79 (52%)		
CAD4TBv5 Score	63 (43, 76)	63 (45, 77)	0.4^4^	85 (70, 93)	86 (71, 97)	0.045^4^	< 0.001^4^
Residency location			0.3^3^			0.3^2^	0.3^2^
Rural	103 (59%)	74 (61%)		184 (58%)	95 (62%)		
Peri-urban	64 (37%)	45 (37%)		114 (36%)	49 (32%)		
Urban	7 (4%)	3 (2%)		20 (6%)	9 (6%)		
Socioeconomic status			0.034^2^			0.4^2^	0.6^2^
Lowest	22 (13%)	18 (15%)		48 (15%)	21 (14%)		
Low	50 (29%)	39 (32%)		91 (29%)	44 (29%)		
Middle	39 (22%)	29 (24%)		73 (23%)	31 (20%)		
High	24 (14%)	15 (12%)		49 (15%)	29 (19%)		
Highest	34 (20%)	17 (14%)		42 (13%)	21 (14%)		
Unknown	5 (3%)	4 (3%)		15 (5%)	7 (5%)		
**II. Cascade of care**							
Attended clinic/hospital (self-reported)		119 (98%)			114 (75%)		< 0.001^3^
Facility attended							< 0.001^3^
Clinic		115 (97%)			26 (23%)		
Hospital		4 (3%)			88 (77%)		
Started TB treatment (self-reported)		115 (94%)			24 (16%)		< 0.001^2^
Started TB treatment (Tier.net)	116 (67%)	88 (72%)	0.3^2^	28 (9%)	15 (10%)	0.7^2^	< 0.001^2^
Completed TB treatment (self-reported)		113 (93%)			22 (14%)		< 0.001^2^
**III. Time to starting and completing TB treatment**							
Time to starting TB treatment (days, self-reported)		21 (14, 29)			42 (31, 73)		< 0.001^4^
Time to starting TB treatment (days, Tier.net)	9 (8, 27)	10 (8, 27)	0.6^4^	41 (33, 74)	49 (38, 73)	0.2^4^	< 0.001^4^
Percentage of individuals starting TB treatment (by self-report) within:							< 0.001^3^
0–30 days		86 (75%)			5 (21%)		
31–60 days		17 (15%)			8 (33%)		
61–90 days		5 (4%)			4 (17%)		
More than 90 days		4 (3%)			5 (21%)		
Unknown		3 (3%)			2 (8%)		
Time to completing TB treatment (days, self-report)		207 (202, 217)			255 (225, 282)		< 0.001^4^

^a^Median (interquartile range—IQR); n (%)

^2^Statistical test used: Pearson’s Chi-squared test

^3^Statistical test used: Fisher’s exact test

^4^Statistical test used: Wilcoxon rank sum test

Baseline age, sex, HIV status, CAD4TBv5 score and socio-demographic characteristics did not differ by symptom status in the microbiologically-confirmed group (Table 2). In the radiologically-suggested TB group, the symptomatic group was older than the asymptomatic group, otherwise baseline characteristics were similar regardless of symptom status (Table 2).

**Table 2:** Baseline characteristics, cascade of care and time to TB treatment commencement and completion among participants who completed the telephonic survey, stratified by TB symptom status at baseline

	**Microbiologically-confirmed TB**			**Radiologically-suggested TB**		
**Characteristic**	**Asymptomatic,** *N* = 103^a^	**Symptomatic**, *N* = 19^a^	***p*-value**	**Asymptomatic**, *N* = 122^a^	**Symptomatic**, *N* = 31^a^	***p*-value**
**I. Baseline Characteristics**						
Age group			0.3^3^			0.039^3^
< 25	13 (13%)	1 (5%)		7 (6%)	1 (3%)	
25–44	38 (37%)	4 (21%)		26 (21%)	1 (3%)	
45–64	31 (30%)	8 (42%)		67 (55%)	19 (61%)	
> 65	21 (20%)	6 (32%)		22 (18%)	10 (32%)	
Sex			0.7^2^			0.7^2^
Male	48 (47%)	8 (42%)		67 (55%)	16 (52%)	
Female	55 (53%)	11 (58%)		55 (45%)	15 (48%)	
HIV ELISA			0.9^2^			0.11^2^
Negative	56 (54%)	10 (53%)		55 (45%)	19 (61%)	
Positive	47 (46%)	9 (47%)		67 (55%)	12 (39%)	
CAD4TBv5 Score	64 (45, 77)	60 (55, 71)	> 0.9^4^	86 (70, 95)	85 (74, 98)	0.6^4^
Residency location			0.4^3^			0.10^3^
Rural	62 (60%)	12 (63%)		71 (58%)	24 (77%)	
Peri-urban	39 (38%)	6 (32%)		42 (34%)	7 (23%)	
Urban	2 (2%)	1 (5%)		9 (7%)	0 (0%)	
Socioeconomic status			0.5^3^			0.3^3^
Lowest	16 (16%)	2 (11%)		15 (12%)	6 (19%)	
Low	29 (28%)	10 (53%)		33 (27%)	11 (35%)	
Middle	26 (25%)	3 (16%)		28 (23%)	3 (10%)	
High	13 (13%)	2 (11%)		22 (18%)	7 (23%)	
Highest	15 (15%)	2 (11%)		18 (15%)	3 (10%)	
Unknown	4 (4%)	0 (0%)		6 (5%)	1 (3%)	
**II. Cascade of care**						
Attended clinic/hospital (self-reported)	101 (98%)	18 (95%)	0.4^3^	90 (74%)	24 (77%)	0.7^2^
Facility attended			0.5^3^			0.6^3^
Clinic	98 (97%)	17 (94%)		22 (24%)	4 (17%)	
Hospital	3 (3%)	1 (6%)		68 (76%)	20 (83%)	
Started TB treatment (self-reported)	98 (95%)	17 (89%)	0.3^3^	19 (16%)	5 (16%)	> 0.9^3^
Started TB treatment (Tier.net)	72 (70%)	16 (84%)	0.2^3^	11 (9%)	4 (13%)	0.5^3^
Completed TB treatment (self-reported)	96 (93%)	17 (89%)	0.3^3^	17 (14%)	5 (16%)	0.8^3^
**III. Time to starting and completing TB treatment**						
Time to starting TB treatment (days, self-reported)	22 (14, 30)	20 (10, 22)	0.12^4^	41 (31, 72)	43 (42, 73)	0.7^4^
Time to starting TB treatment (days, Tier.net)	9 (8, 24)	26 (11, 29)	0.14^4^	45 (34, 58)	66 (55, 79)	0.2^4^
Percentage of individuals starting TB treatment (by self-report) within:			0.6^3^			> 0.9^3^
0–30 days	72 (73%)	14 (82%)		4 (21%)	1 (20%)	
31–60 days	16 (16%)	1 (6%)		6 (32%)	2 (40%)	
61–90 days	4 (4%)	1 (6%)		3 (16%)	1 (20%)	
More than 90 days	4 (4%)	0 (0%)		4 (21%)	1 (20%)	
Unknown	2 (2%)	1 (6%)		2 (11%)	0 (0%)	
Time to completing TB treatment (days, self-report)	208 (202, 218)	205 (202, 216)	0.3^4^	259 (224, 292)	255 (225, 257)	0.7^4^

^a^Median (IQR); n (%)

^2^Statistical test used: Pearson’s Chi-squared test

^3^Statistical test used: Fisher’s exact test

^4^Statistical test used: Wilcoxon rank sum test

### Cascade of care

**Fig. 3:**
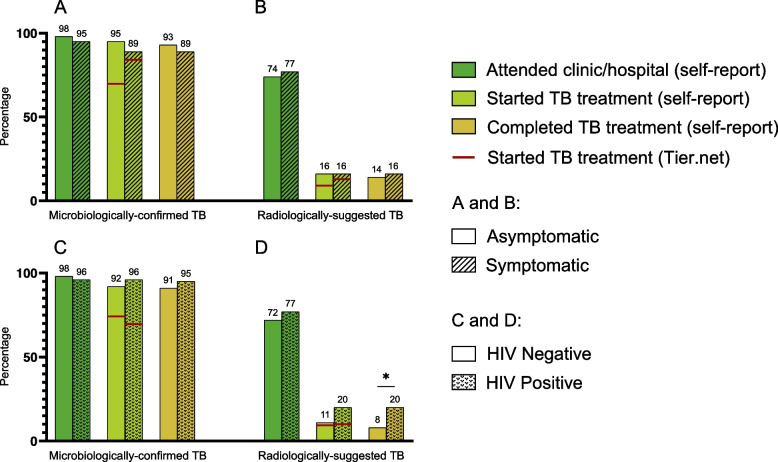
Cascade of care for (**A**) microbiologically-confirmed TB, stratified by symptom status, (**B**) radiologically-suggested TB, stratified by symptom status, (**C**) microbiologically-confirmed TB, stratified by HIV status and (**D**) radiologically-suggested TB, stratified by HIV status. * = *p*-value < 0.05

In the microbiologically-confirmed TB group, 119/122 (98%) reported linking to care by having attended a health care facility as a result of their referral, after which 115/122 (94%) started TB treatment and 113/122 (93%) completed TB treatment (Table 1 Part II). Among people with microbiologically-confirmed TB, people who were asymptomatic at baseline and thus met the study definition for subclinical TB completed the steps along the cascade of care at high rates [98% (101/103) linked to care, 95% (98/103) started TB treatment, 93% (96/103) completed TB treatment] and these high rates did not significantly differ from people with symptomatic microbiologically-confirmed TB [95% (18/19) linked to care, 89% (17/19) started TB treatment, 89% (17/19) completed TB treatment] (Fig. 3A, Table 2 Part II). HIV status at baseline was not associated with any significant differences in percentage attending initial referral sites, starting TB treatment or completing TB treatment in the microbiologically-confirmed TB group (Fig. 3C, Additional file 1: Table S1 Part I).

Compared to people with microbiologically-confirmed TB, fewer people in the radiologically-suggested TB group attended a health care facility as a result of their referral (114/153 (75%), p < 0.001), after which far fewer started TB treatment (24/153 (16%), p < 0.001) and completed TB treatment (22/153 (14%), p < 0.001). Symptom status at baseline was not associated with any significant differences in percentage attending initial referral sites, starting or completing TB treatment in the radiologically-suggested TB group (Fig. 3B, Table 2). In this group, a higher percentage of PLWH completed TB treatment compared to their HIV negative counterparts (20% vs 8%, p = 0.032, Fig. 3D, Additional file 1: Table S1 Part I).

Self-reported time to starting treatment also differed between the two groups: those with microbiologically-confirmed TB group started earlier than those with radiologically-suggested TB (median times of 21 days (IQR 14–29) and 42 days (IQR 31–73), respectively, p < 0.001, Table 1, Part III). Within each group, time to starting treatment did not differ depending on baseline symptom or HIV status. Due to different referral pathways, the health care facility visited after referral differed between the microbiologically-confirmed and radiologically-suggested groups: of the 119 individuals who attended a referral visit in the microbiologically-confirmed TB group, 115/119 (97%) attended a local primary health care clinic as their initial referral site and 4/119 (3%) attended Hlabisa District Hospital, whereas only 26/114 (23%) of the individuals who attended a referral visit in the radiologically-suggested TB group attended a local primary health care clinic and 88/114 (77%) attended Hlabisa District Hospital. Within both groups, attendance at clinic or hospital as initial referral site did not differ depending on baseline symptom or HIV status.

### Health systems data

Data obtained from the national Tier.net database showed that more patients with microbiologically-confirmed TB than those with radiologically-suggestive TB had a matching electronic record that documented starting of TB treatment (88/122 (72%) vs. 15/153 (10%), p < 0.001, Table 1, Part II). According to Tier.net data, time to TB treatment commencement was shorter in the microbiologically-confirmed group (10 days (IQR 8–27)), compared with the radiologically-suggested group (49 days (IQR 38–73), p < 0.001). These data, which corresponded to people who completed the telephonic survey, did not show any significant differences in TB treatment commencement rates or days to TB treatment commencement when stratified by baseline symptom status or HIV status (Table 2, Part II, Additional file 1: Table S1 Part II). When the health system data analysis was expanded to those who were eligible regardless of whether they participated in the follow-up survey, the rates of TB treatment commencement (116/174 (67%) in the microbiologically-confirmed TB group and 28/318 (9%) in the radiologically-suggested TB group) and days to TB treatment commencement (median 9 days (IQR 8–27) in microbiologically-confirmed TB group and median 41 days (IQR 33–74) in the radiologically-suggested TB group) did not differ significantly from rates from the subgroup that participated in the follow-up survey (Table 1, Parts II & III). Treatment commencement rates also did not differ between the subclinical TB group and those with symptomatic microbiologically-confirmed TB in the larger eligible group (94/143 (66%) vs. 22/31 (71%), p = 0.6).

## Discussion

In a community-based TB screening program in rural KwaZulu-Natal that referred people with microbiologically-confirmed and radiologically-suggested TB to routine health services, we found high rates of linkage to care, TB treatment commencement and TB treatment completion among people with microbiologically-confirmed TB, despite the fact that over 80% of people in this group had no symptoms at baseline, consistent with subclinical TB. Among people with chest x-ray findings consistent with active TB but no microbiological confirmation, rates of linkage to care were lower. In both groups, links along the cascade of care, whether measured by self-report or linkage to the health system electronic record, did not differ by baseline symptom status. The results of this study highlight that participants in community-based TB screening may be highly willing to accept referrals, start and complete treatment for microbiologically-confirmed subclinical TB.

Subclinical TB is increasingly being recognized as an important part of the TB pandemic [5, 7, 15, 16]. A recent systematic review by Frascella et al. showed that a median of 50.4% of microbiologically-confirmed TB cases was subclinical, based on prevalence surveys conducted globally [5]. Similarly, South Africa’s First National TB Prevalence Survey from 2021 showed that 57.8% of participants with microbiologically-proven TB did not report any symptoms [17]. Despite this, real-world TB screening programmes emphasize TB symptoms for selecting those for sputum testing. The symptom-based approach would therefore miss a large proportion of prevalent TB cases. Active case-finding (ACF) approaches which do not focus on symptoms are therefore increasingly important [18], and people’s willingness to link to care and complete TB treatment when diagnosed with asymptomatic TB during screening programmes will determine the impact of these programs.

This study used two complementary methods to assess linkage to care. In those participants whom we were able to re-contact by telephone approximately 3 years after the initial screening, we obtained detailed cascade of care information; by this method, people with microbiologically-confirmed subclinical TB reported very high rates of linkage to care (98%), TB treatment commencement (95%) and completion (93%). These numbers should be considered a “best possible” estimate due to several sources of bias that may have inflated linkage rates: survivor bias (eligible individuals who had died or otherwise been lost to follow-up were excluded from this study component), recall bias (difficulty accurately remembering events of 3 years prior) and social desirability bias (perceived pressure to report positive results to please the interviewer). Use of health system linkage data for the entire eligible group, including those who had died and been lost-to-follow-up, found evidence that 66% of people with microbiologically-confirmed subclinical TB commenced TB treatment (the only care cascade measure reliably recorded in the database). This estimate should be considered a “worst possible” estimate because incomplete or incorrect data entry into the electronic TB registry and imperfect performance of matching algorithms may have resulted in an undercounting of people who actually started TB treatment. If the true number of people with microbiologically-confirmed subclinical TB who commenced TB treatment likely lies between 66 and 95%, the proportion of persons starting TB treatment in this study was higher or as high as rates that have been reported in other ACF studies recently conducted in Southern Africa, where it ranged from 51 to 93% [19–23]. Possible explanations for the high rates of linkage in our study is the longstanding relationship that AHRI has with the local community, which has resulted in trust between the two parties, and the establishment of a memorandum of understanding between the study and the local DoH regarding clinical use of study screening results prior to commencement of the study.

Linkage to care after a positive test during community-based screening differed between the microbiologically-confirmed and radiologically-suggested groups (98% vs 75%). This difference is most likely due to the difference in referral sites, with local standards of care requiring that most individuals with negative sputum microbiology but chest x-rays suggestive of TB be referred to the district hospital for evaluation by a medical doctor rather than referral to primary health care clinics, where care is provided by nurses. Since the hospital is farther away, requiring added transport costs and time, this could have accounted in part for the number of participants that did not link to the referral centre in this group. A study in Zimbabwe found the opposite result in their population, with a larger percentage of their microbiologically-confirmed group being lost to follow-up before treatment initiation than their clinically diagnosed group [24]. This suggests that the difference in linkage to care that we see may be due to logistical barriers to care as described above rather than the basis of diagnosis.

The largest difference between the care cascades of the two groups was how many started treatment after their referral visits. Treatment initiation in the microbiologically-confirmed group was very high at 94%, regardless of the patient’s symptom status. In the radiologically-suggested group, only 16% of participants initiated TB treatment. It is expected that there would be a difference between the two groups – in those individuals who made it to care, the treating clinicians could have rightfully decided that there was not enough evidence to start TB treatment, since many other infectious and non-infectious diseases can mimic TB’s x-ray findings [25]. It is, however, possible that some of these participants did have TB disease and other factors played a role in them not initiating treatment. The complexity of the clinical approach to managing individuals with only radiological signs of TB and the extra steps in the referral pathway (travel distance to the hospital, repeat sputum or other further investigations, and/or trial of antibiotics and follow-up), could have led to some participants being lost to follow-up. These extra steps also explain the difference in time to treatment initiation between the two groups – since the microbiologically-confirmed group had fewer steps in their referral pathway, they could start treatment earlier. This difference was also noticed in a recent ACF study in Peru [26]. Studies have also shown that x-ray interpretation in an ACF setting differs markedly depending on the level of training of the interpreter, and therefore the treating clinician at the hospital could have disagreed with the radiologist’s interpretation of the x-ray [27, 28]. A more appropriate outcome measure for this group would have been the quality of diagnostic and clinical care these participants received rather than a treatment initiation percentage, but this is complicated to assess and falls outside the scope of this paper.

While there were many differences in the care cascades between the two study groups, baseline symptom status was not associated with significant differences within either group. We initially anticipated that symptom status would alter the treatment cascade, with asymptomatic people being less likely to follow up their referral and/or start and complete treatment, but that was not the case. This is an important finding since little is known about people’s willingness to start treatment when they do not have symptoms. This question will become even more relevant as TB screening programmes are rolled out more broadly, as recommended by the WHO’s operational handbook on tuberculosis [29]. Other ACF studies have also found no difference in linkage to care between symptomatic and asymptomatic participants [22, 24, 30], but Ananda et al. reported that although they found no difference in treatment initiation between the two groups, their symptomatic group had better treatment outcomes [30].

Baseline HIV status was also not associated with any significant differences in the microbiologically-confirmed TB group, but in the radiologically-suggested group PLWH commenced and completed TB treatment more frequently than their HIV-negative counterparts. Although the reason for this difference was not apparent in our data, it is possible that people living with HIV are better established in primary health care at the clinic level or that local clinicians have a lower threshold for commencing TB treatment in a person living with HIV who has a suggestive chest x-ray.

In addition to the sources of bias discussed above, this study had other limitations. Conducting telephonic surveys has many inherent challenges, including failure to reach all eligible people. This can be due to a wide variety of reasons, such as changed phone numbers, death, imprisonment, and people being unable to take calls during working hours. Unfortunately, the reasons for survey non-response potentially introduced bias. Additionally, refusal to be surveyed could have been because of a negative experience during the initial population-based survey; such bias may have led to overestimation of linkage to care and TB treatment commencement. There was a significant difference in response rates between the microbiologically-confirmed and radiologically-suggested groups. One reason for this is that individuals eligible for the microbiologically-confirmed TB group were also eligible for another study at AHRI; therefore, at the time of this study, their contact telephone numbers were more up-to-date than those individuals eligible for the radiologically-suggestive TB group. The higher response rates in the microbiologically-confirmed group is a source of potential bias and overestimate the differences between the two groups. The sampled group could also be biased towards health-seeking behaviour compared to the general population, since they form part of the approximately 50% of the population that participated in the initial population-based survey [11]. An additional limitation is that our study was only based in one district and specific features of our study population limits generalizability. These features include the high HIV prevalence and the longstanding relationship between AHRI and the community, and AHRI and the DoH. We also do not have additional information regarding what further examinations and investigations the radiologically-suggested group underwent after referral, which could have determined which participants were advised to start TB treatment. For this reason, we limited our study definition of subclinical TB to asymptomatic individuals with microbiological confirmation of TB. Limitations related to the Tier.net system are highlighted above, and in addition we only had access to the Tier.net data until March 2020 and therefore data regarding TB treatment may have been incomplete.

## Conclusions

Clear guidelines do not yet exist to treat subclinical TB and there is concern that even once such guidelines exist people who screen positive for TB in the absence of symptoms may be unwilling to accept TB treatment. We have shown that the majority of people diagnosed with microbiologically-proven subclinical TB during a population-based survey in rural KwaZulu-Natal, South Africa, were willing to start TB treatment despite the lack of symptoms. Design of decentralized diagnostic and care pathways for patients received from community-based screening programs, especially for patients who have a positive screening chest x-ray and negative sputum are urgently needed to increase access to quality care and decrease barriers to TB treatment, when clinically warranted. Further research is needed in other regions to determine if linkage to care in asymptomatic people is similarly high in other settings, or if our high rates of linkage are a result of the research organisation’s strong relationship with the community. Further research is also needed to better understand the benefits and potential harms of community-based screening. Subclinical TB forms a large part of the global TB burden, and therefore improving the experience of people diagnosed with subclinical TB during active case-finding will be an important part of future efforts to end TB in our lifetime.

### Supplementary Information

**Additional file 1. **Contains the following supplementary materials: list of Vukuzazi team members, supplementary methods, and Table S1 (Cascade of care and time to TB treatment commencement and completion among participants who completed the telephonic survey, stratified by HIV status at baseline).

## Data Availability

De-identified data can be accessed via the Africa Health Research Institute Data Repository. Interested parties can email RDMServiceDesk@ahri.org to request access, subject to data access agreement.

## Abbreviations

TB: Tuberculosis
Mtb: *Mycobacterium tuberculosis*
WHO: World Health Organization
HIV: Human immunodeficiency virus
PLWH: People living with HIV
AHRI: Africa Health Research Institute
DSA: Demographic surveillance area
WHO-4SS: WHO 4-symptom screening
ADS-TB: Abnormality detected, significant-tuberculosis
DoH: Department of Health
MOU: Memorandum of understanding
NHLS: National Health Laboratory Service
ID number: Identification number
POPIA: Protection of Personal Information Act
IQR: Interquartile range
ACF: Active case-finding

## References

[1] Global tuberculosis report 2022. Geneva: World Health Organization; 2022. licence: CC BY-NC-SA 3.0 IGO.

[2] Straetemans M, Bierrenbach AL, Nagelkerke N, Glaziou P, van der Werf MJ (2010). The effect of tuberculosis on mortality in HIV positive people: A meta-analysis. PLoS ONE..

[3] Subbaraman R, Jhaveri T, Nathavitharana RR (2020). Closing gaps in the tuberculosis care cascade: An action-oriented research agenda. Journal of Clinical Tuberculosis and Other Mycobacterial Diseases.

[4] World Health Organization (2013). Systematic screening for active tuberculosis: principles and recommendations.

[5] Frascella B, Richards AS, Sossen B, Emery JC, Odone A, Law I (2020). Subclinical Tuberculosis Disease—a review and analysis of prevalence surveys to inform definitions, burden, associations, and screening methodology. Clin Infect Dis..

[6] Esmail H, Dodd PJ, Houben RM (2018). Tuberculosis transmission during the subclinical period: Could unrelated cough play a part?. Lancet Respir Med.

[7] Wong EB (2021). It Is Time to Focus on Asymptomatic Tuberculosis. Clin Infect Dis.

[8] National Department of Health, South Africa. National tuberculosis management guidelines 2014. Pretoria: National Department of Health; 2014. https://knowledgehub.health.gov.za/elibrary/national-tuberculosis-managementguidelines Accessed 24 January 2024.

[9] Tuberculosis Coalition for Technical Assistance. International Standards for Tuberculosis Care (ISTC). The Hague: Tuberculosis Coalition for Technical Assistance. 2006.

[10] Gunda R, Koole O, Gareta D, Olivier S, Surujdeen A, Smit T (2021). Cohort profile: The vukuzazi (‘wake up and know yourself’ in isizulu) population science programme. Int J Epidemiol..

[11] Wong EB, Olivier S, Gunda R, Koole O, Surujdeen A, Gareta D (2021). Convergence of infectious and non-communicable disease epidemics in rural South Africa: A cross-sectional, population-based multimorbidity study. Lancet Glob Health..

[12] Anthony T, Ayles H, Beyers N, Bierrenbach A, Birdthistle I, Bloss E (2011). Tuberculosis prevalence surveys: A Handbook.

[13] Fehr J, Gunda R, Siedner MJ, Hanekom W, Ndung´u T, Grant A (2023). CAD4TB software updates: Different triaging thresholds require caution by users and regulation by authorities. Int J Tuberc Lung Dis..

[14] Fehr J, Konigorski S, Olivier S, Gunda R, Surujdeen A, Gareta D (2021). Computer-aided interpretation of chest radiography reveals the spectrum of tuberculosis in rural South Africa. NPJ Digit Med.

[15] Kendall EA, Shrestha S, Dowdy DW (2021). The epidemiological importance of subclinical tuberculosis. A critical reappraisal. Am J Resp Crit Care Med..

[16] Esmail H, Macpherson L, Coussens AK, Houben RMGJ (2022). Mind the gap – managing tuberculosis across the disease spectrum. eBioMedicine.

[17] Moyo S, Ismail F, Van der Walt M, Ismail N, Mkhondo N, Dlamini S (2022). Prevalence of bacteriologically confirmed pulmonary tuberculosis in South Africa, 2017–19: A multistage, cluster-based, cross-sectional survey. Lancet Infect Dis.

[18] Pai M, Dewan PK, Swaminathan S (2023). Transforming tuberculosis diagnosis. Nat Microbiol.

[19] Shapiro AE, van Heerden A, Schaafsma TT, Hughes JP, Baeten JM, van Rooyen H (2018). Completion of the tuberculosis care cascade in a community-based HIV linkage-to-care study in South Africa and Uganda. J Int AIDS Soc..

[20] Naidoo K, Moodley MC, Hassan-Moosa R, Dookie N, Yende-Zuma N, Perumal R (2022). Recurrent subclinical tuberculosis among antiretroviral therapy–accessing participants: Incidence, clinical course, and outcomes. Clin Infect Dis.

[21] Maraba N, Chihota V, McCarthy K, Churchyard GJ, Grant AD (2018). Linkage to care among adults being investigated for tuberculosis in South Africa: pilot study of a case manager intervention. BMJ Open.

[22] Bassett IV, Forman LS, Govere S, Thulare H, Frank SC, Mhlongo B (2019). Test and treat TB: A pilot trial of GeneXpert MTB/Rif screening on a mobile HIV testing unit in South Africa. BMC Infect Dis..

[23] Martinson NA, Lebina L, Webb EL, Ratsela A, Varavia E, Kinghorn A (2021). Household contact tracing with intensified tuberculosis and human immunodeficiency virus screening in South Africa: A cluster-randomized trial. Clin Infect Dis.

[24] Sengai T, Timire C, Harries AD, Tweya H, Kavenga F, Shumba G (2019). Mobile targeted screening for tuberculosis in Zimbabwe: Diagnosis, linkage to care and treatment outcomes. Public Health Action.

[25] Davies PD, Pai M (2008). The diagnosis and misdiagnosis of tuberculosis. Int J Tuberc Lung Dis.

[26] Yuen CM, Puma D, Millones AK, Galea JT, Tzelios C, Calderon RI (2021). Identifying barriers and facilitators to implementation of community-based tuberculosis active case finding with mobile X-ray units in Lima, Peru: A re-aim evaluation. BMJ Open..

[27] Henostroza G, Harris JB, Kancheya N, Nhandu V, Besa S, Musopole R (2016). Chest radiograph reading and recording system: Evaluation in frontline clinicians in Zambia. BMC Infectious Diseases..

[28] Timire C, Sandy C, Ngwenya M, Woznitza N, Kumar AM, Takarinda KC (2019). Targeted active screening for tuberculosis in Zimbabwe: Are Field Digital chest X-ray ratings reliable?. Public Health Action..

[29] WHO operational handbook on tuberculosis. Module 2: screening - systematic screening for tuberculosis disease. Geneva: World Health Organization; 2021. Licence: CC BY-NC-SA 3.0 IGO. 33822560

[30] Ananda NR, Triasih R, Dwihardiani B, Nababan B, Hidayat A, Chan G (2023). Spectrum of TB disease and treatment outcomes in a mobile community based active case finding program in Yogyakarta Province, Indonesia. Trop Med Infect Dis..

