# Supplementary material for: Subclinical tuberculosis linkage to care and completion of treatment following community-based screening in rural South Africa
Source: BMC Glob Public Health. 2024 Jun 2;2:30. doi: 10.1186/s44263-024-00059-0 (PMC11144138; doi:10.1186/s44263-024-00059-0)
Supplement: Supplementary file 1 — Additional file 1. Contains the following supplementary materials: list of Vukuzazi team members, supplementary methods, and Table S1 (Cascade of care and time to TB treatment commencement and completion among participants who completed the telephonic survey, stratified by HIV status at baseline). [file 44263_2024_59_MOESM1_ESM.docx]

**Additional file 1**

**Table of contents**

1. List of Vukuzazi team members page 1-3

2. Supplementary methods page 4-5

3. Supplementary tables page 6

4. References page 7

**List of Vukuzazi team members**

The following people contributed significantly to the implementation of the Vukuzazi study:^1^

| **Name** | **Role** |
| --- | --- |
| Deenan Pillay | Principal investigator |
| Willem Hanekom | Principal investigator |
| Emily Wong | Co-principal investigator |
| Mark Siedner | Co-principal investigator |
| Olivier Koole | Co-principal investigator |
| Thumbi Ndung’u | Co-investigator |
| Thandeka Khoza | Co-investigator |
| Kobus Herbst | Co-investigator |
| Kathy Baisley | Co-investigator |
| Janet Seeley | Co-investigator |
| Alison Grant | Co-investigator |
| Resign Gunda | Programme manager |
| Ashmika Surujdeen | Study coordinator |
| Theresa Smit | Head: Diagnostic research |
| Dickman Gareta | Head: Research data management |
| Day Munatsi | Head: Research data systems |
| Ngcebo Mhlongo | Study physician |
| Sanah Bucibo | Lead nurse |
| Tshwaraganang Modise | Research data manager |
| Stephen Olivier | Statistician |
| Gregory Ording-Jespersen | Laboratory data supervisor |
| Innocentia Mpofana | Diagnostic laboratory manager |
| Jaco Dreyer | Senior research data manager |
| Siyabonga Nxumalo | Research data manager |
| Khadija Khan | Biorepository manager |
| Zizile Sikhosana | Somkhele laboratory supervisor |
| Sashen Moodley | Microbiology laboratory supervisor |
| Hollis Shen | Head: Exploratory research division |
| Kennedy Nyamande | Pulmonology consultant |
| Mosa Suleman | Pulmonology consultant |
| Jaikrishna Kalideen | Radiologist |
| Ramesh Jackpersad | Radiologist |
| Kgaugelo Moropane | Radiographer |
| Boitsholo Mfolo | Radiographer |
| Khabonina Malomane | Radiographer |
| Hlolisile Khumalo | Nursing manager |
| Nompilo Buthelezi | Training coordinator |
| Nozipho Mbonambi | Professional nurse |
| Hloniphile Ngubane | Professional nurse |
| Thokozani Simelane | Professional nurse |
| Khanyisani Buthelezi | Professional nurse |
| Sphiwe Ntuli | Professional nurse |
| Nombuyiselo Zondi | Professional nurse |
| Siboniso Nene | Professional nurse |
| Bongumenzi Ndlovu | Enrolled nurse |
| Talente Ntimbane | Enrolled nurse |
| Mbali Mbuyisa | Enrolled nurse |
| Xolani Mkhize | Enrolled nurse |
| Melusi Sibiya | Enrolled nurse |
| Ntombiyenkosi Ntombela | Enrolled nurse |
| Mandisi Dlamini | Enrolled nurse |
| Hlobisile Chonco | Enrolled nurse |
| Hlengiwe Dlamini | Enrolled nurse |
| Doctar Mlambo | Enrolled nurse |
| Nonhlanhla Mzimela | Enrolled nurse |
| Zinhle Buthelezi | Enrolled nurse |
| Zinhle Mthembu | Enrolled nurse |
| Thokozani Bhengu | Enrolled nurse |
| Sandile Mthembu | Enrolled nurse |
| Phumelele Mthethwa | Enrolled nurse |
| Zamashandu Mbatha | Enrolled nurse |
| Welcome Petros Mthembu | Enrolled nurse |
| Anele Mkhwanazi | Clinical research assistant supervisor |
| Mandlakayise Zikhali | Clinical research assistant supervisor |
| Phakamani Mkhwanazi | Clinical research assistant |
| Ntombiyenhlanhla Mkhwanazi | Clinical research assistant |
| Rose Myeni | Clinical research assistant |
| Fezeka Mfeka | Clinical research assistant |
| Hlobisile Gumede | Clinical research assistant |
| Nonceba Mfeka | Clinical research assistant |
| Ayanda Zungu | Clinical research assistant |
| Hlobisile Gumede | Clinical research assistant |
| Nonhlanhla Mfekayi | Clinical research assistant |
| Smangaliso Zulu | Clinical research assistant |
| Mzamo Buthelezi | Clinical research assistant |
| Senzeni Mkhwanazi | Clinical research assistant |
| Mlungisi Dube | Clinical research assistant |
| Philippa Mathews | Clinical governance |
| Siphephelo Dlamini | AHRI nursing manager |
| Hosea Kambonde | IT Systems Developer |
| Lindani Mthembu | Information technology assistant |
| Seneme Mchunu | Information technology assistant |
| Sibahle Gumbi | Research admin assistant |
| Tumi Madolo | Research data manager |
| Thengokwakhe Nkosi | Driver |
| Sibusiso Mkhwanazi | Driver |
| Sibusiso Nsibande | Driver |
| Mpumelelo Steto | Driver |
| Sibusiso Mhlongo | Driver |
| Velile Vellem | Driver |
| Pfarelo Tshivase | Driver |
| Jabu Kwinda | Driver |
| Bongani Magwaza | General worker |
| Siyabonga Nsibande | General worker |
| Skhumbuzo Mthombeni | General worker |
| Sphiwe Clement Mthembu | General worker |
| Antony Rapulana | Laboratory technologist |
| Jade Cousins | Laboratory technologist |
| Thabile Zondi | Laboratory technologist |
| Nagavelli Padayachi | Laboratory technologist |
| Freddy Mabetlela | Laboratory technologist |
| Simphiwe Ntshangase | Laboratory technician/LIMS administrator |
| Nomfundo Luthuli | Laboratory technician |
| Sithembile Ngcobo | Laboratory technologist |
| Kayleen Brien | Laboratory technologist |
| Sizwe Ndlela | Laboratory technician |
| Nomfundo Ngema | Laboratory technician |
| Nokukhanya Ntshakala | Laboratory technician |
| Anupa Singh | Laboratory technician |
| Rochelle Singh | Laboratory technician |
| Logan Pillay | Laboratory technician |
| Kandaseelan Chetty | Laboratory technician |
| Ashentha Govender | Laboratory technician |
| Pamela Ramkalawon | Laboratory research technician |
| Nondumiso Mabaso | Laboratory intern |
| Kimeshree Perumal | Laboratory intern |
| Senamile Makhari | Biorepository laboratory technician |
| Nondumiso Khuluse | Biorepository laboratory technician |
| Nondumiso Zitha | Biorepository research assistant |
| Hlengiwe Khathi | Biorepository research assistant |
| Mbuti Mofokeng | Clinical specimen driver/Laboratory assistant |
| Nomathamsanqa Majozi | Public engagement |
| Nceba Gqaleni | Public engagement |
| Hannah Keal | Communications |
| Phumla Ngcobo | Communications |
| Costa Criticos | Operational oversight |
| Raynold Zondo | Operational oversight |
| Dilip Kalyan | Operational oversight |
| Clive Mavimbela | Operational oversight |
| Anand Ramnanan | Procurement |
| Sashin Harilall | Grants office |

**Supplementary methods**

**X-ray categorization**

X-rays were categorized by the radiologist according to the WHO Tuberculosis Prevalence Surveys handbook’s guidelines for interpretation at a central level.^2^ According to this classification system, an x-ray can fall into one of the following categories, copied from the handbook:

“Normal. No abnormality is detected on the chest X-ray. Normal anatomical variants and frequent degenerative findings (such as Azygos lobe, unfolded aortic arch, degenerative vascular calcification, prominent superior vena cava, cervical rib, and accessory or hypoplastic ribs) are considered normal and classified in this category.

Abnormality detected – not significant. Chest X-ray images where the radiologist detects some abnormality but is convinced of its clinical insignificance from the point of view of pulmonary TB. Examples include bone fracture (any type), scoliosis or kyphosis, extrapulmonary soft tissue masses such as goitre, dextrocardia, abnormal cardiac contour, signs of mitral stenosis, aortic aneurysm and abnormalities of pulmonary vasculature. To avoid complications, abnormal cardiac size (cardiomegaly, or enlargement of any particular chamber) is also classified in this category, since it bears no relation to pulmonary TB and also because in isolation it is a poor indictor of cardiac disease in the population.

Abnormality detected, significant – no active disease. Chest X-ray images that show significant abnormality but the radiologist is certain that they do not point to any active disease. Examples include pleural thickening, evidence of prior surgery such as lobectomy or pneumonectomy, classical fibrosis, residual and/or calcified scars and densely calcified nodules without any peripheral satellite lesions.

Abnormality detected, significant – not tuberculosis. Chest X-ray images where significant abnormality is detected but the radiologist is certain that the cause is non-tubercular. Examples include emphysema, classic bronchiectasis, classic lobar consolidation with air bronchograms (conventionally labelled as bacterial pneumonia), spiculated or stellate masses (which suggest neoplastic nature), canon ball metastases and pulmonary congestion or other vascular abnormalities.

Abnormality detected, significant – tuberculosis. Chest X-ray images that show abnormalities usually associated with pulmonary TB. Examples include cavitation, apical involvement, parenchymal opacities with or without pleural effusion, parenchymal opacities with mediastinal or hilar lymph node enlargement, isolated lymphadenopathy, diagonal parenchymal involvement, miliary parenchymal mottling, and involvement of typical tubercular sites such as apices and upper segments of lower lobes. Although not typical of TB, isolated pleural effusion and pneumothorax are included here. All cases where the radiologist, during the course of usual practice, would have noted the impression using terms such as “suggestive of TB”, “consistent with TB”, “most likely TB”, “most probably TB”, “probably TB” (that is, where the radiologist is reasonably sure that the etiological cause is TB but is hesitant to volunteer a diagnosis in writing) should be classified in this category.

Abnormality detected, significant – unclassified. Chest X-ray images where significant abnormality is detected but the radiologist is not sure if the etiology is tubercular or nontubercular. Example could be multiple non-homogenous nodular opacities, bizarre patterns etc. It is emphasized that this category be reserved for cases where considerable uncertainty exists, and not be used for ‘convenience’ sake.”

**Supplementary tables**

**Table S1: Cascade of care and time to TB treatment commencement and completion among participants who completed the telephonic survey, stratified by HIV status at baseline**

|  | **Microbiologically-confirmed TB** | | | **Radiologically-suggested TB** | | |
| --- | --- | --- | --- | --- | --- | --- |
| **Characteristic** | **HIV negative**,  N = 66^1^ | **HIV positive**,  N = 56^1^ | **p-value** | **HIV negative**,  N = 74^1^ | **HIV positive**,  N = 79^1^ | **p-value** |
| **I. Cascade of care** |  |  |  |  |  |  |
| Attended clinic/hospital (self-reported) | 65 (98%) | 54 (96%) | 0.6^3^ | 53 (72%) | 61 (77%) | 0.4^2^ |
| Facility attended |  |  | 0.3^3^ |  |  | 0.067^2^ |
| Clinic | 64 (98%) | 51 (94%) |  | 8 (15%) | 18 (30%) |  |
| Hospital | 1 (2%) | 3 (6%) |  | 45 (85%) | 43 (70%) |  |
| Started TB treatment (self-reported) | 61 (92%) | 54 (96%) | 0.5^3^ | 8 (11%) | 16 (20%) | 0.11^2^ |
| Started TB treatment (Tier.net) | 49 (74%) | 39 (70%) | 0.6^2^ | 7 (9%) | 8 (10%) | 0.9^2^ |
| Completed TB treatment (self-reported) | 60 (91%) | 53 (95%) | 0.5^3^ | 6 (8%) | 16 (20%) | 0.032^2^ |
| **II. Time to starting and completing TB treatment** |  |  |  |  |  |  |
| Time to starting TB treatment (days, self-report)^3^ | 19 (13, 27) | 22 (19, 31) | 0.13^4^ | 37 (32, 42) | 56 (29, 121) | 0.3^4^ |
| Time to starting TB treatment (days, Tier.net) | 10 (8, 27) | 10 (8, 29) | 0.8^4^ | 42 (34, 55) | 63 (47, 79) | 0.2^4^ |
| Percentage of individuals starting TB treatment (by self-report) within: |  |  | 0.8^3^ |  |  | 0.3^3^ |
| 0-30 days | 47 (77%) | 39 (72%) |  | 1 (13%) | 4 (25%) |  |
| 31-60 days | 7 (11%) | 10 (19%) |  | 4 (50%) | 4 (25%) |  |
| 61-90 days | 3 (5%) | 2 (4%) |  | 1 (13%) | 3 (19%) |  |
| More than 90 days | 2 (3%) | 2 (4%) |  | 0 (0%) | 5 (31%) |  |
| Unknown | 2 (3%) | 1 (2%) |  | 2 (25%) | 0 (0%) |  |
| Time to completing TB treatment (days, self-report) | 206 (198, 220) | 209 (204, 217) | 0.2^4^ | 238 (225, 260) | 257 (227, 320) | 0.2^4^ |
| ^1^Median (IQR); n (%) | | | | | | |
| ^2^Statistical test used: Pearson’s Chi-squared test | | | | | | |
| ^3^Statistical test used: Fisher’s exact test | | | | | | |
| ^4^Statistical test used: Wilcoxon rank sum test | | | | | | |

**References**

1. Wong EB, Olivier S, Gunda R, Koole O, Surujdeen A, Gareta D, et al. Convergence of infectious and non-communicable disease epidemics in rural South Africa: A cross-sectional, population-based multimorbidity study. The Lancet Global Health. 2021;9(7). doi:10.1016/s2214-109x(21)00176-5

2. Anthony T, Ayles H, Beyers N, Bierrenbach A, Birdthistle I, Bloss E, et al. Tuberculosis prevalence surveys: A Handbook. Geneva: World Health Organization; 2011.
